# The Effects of MicroRNAs on Key Signalling Pathways and Epigenetic Modification in Fibroblast-Like Synoviocytes of Rheumatoid Arthritis

**DOI:** 10.1155/2018/9013124

**Published:** 2018-05-10

**Authors:** Wenming Hong, Pengying Zhang, Xinming Wang, Jiajie Tu, Wei Wei

**Affiliations:** ^1^Institute of Clinical Pharmacology, Key Laboratory of Anti-Inflammatory and Immune Medicine, Ministry of Education, Anhui Collaborative Innovation Center of Anti-Inflammatory and Immune Medicine, Anhui Medical University, Hefei, China; ^2^Department of Neurosurgery, First Affiliated Hospital of Anhui Medical University, Hefei, China

## Abstract

MicroRNAs (miRNAs) are small noncoding RNAs that regulate gene expression at the posttranscriptional level via direct binding to the 3′-untranslated region (UTR) of target mRNAs. Emerging evidence shows that miRNAs play crucial roles in controlling and modulating immune system-related diseases. This review focuses on the role played by miRNAs in fibroblast-like synoviocytes (FLS), which is a key cellular component within synovia, during the establishment and maintenance of rheumatoid arthritis (RA), a systemic inflammatory autoimmune disease. It also provides an overview and classification of known functional miRNAs in RA FLS and summarizes the potential uses of these small molecules in RA diagnosis and treatment.

## 1. Background

Rheumatoid arthritis (RA) is a systemic inflammatory autoimmune disease [1], which affects approximately 1% of the world population. The male : female ratio for RA is nearly 1 : 3. RA is mainly diagnosed by clinical features and some validated lab values [2, 3], such as blood tests for rheumatoid factor (RF) and anticitrullinated protein antibodies (ACPA), erythrocyte sedimentation rate, and C-reactive protein; radiography for the damage to cartilage, tendons, and bones; and magnetic resonance imaging (MRI) for synovial inflammation. Currently, RA is not curable. Physical, occupational, and nutritional therapies are three main nonpharmacological approaches for treatment of RA, and some anti-inflammatory drugs and analgesia are also used to repress its symptoms. Recently, with the deeper understanding obtained regarding the molecular pathogenesis of RA, disease-modifying anti-RA drugs (DMARDs) have been developed to treat RA [4]. Except conventional DMARDs, novel biological DMARDs, including tocilizumab, certolizumab, etanercept, adalimumab, anakinra, abatacept, infliximab, rituximab, and golimumab, regulate different specific cellular components or targets in immune system and correspondingly yield significant improvement of RA and the patient outcomes [5]. For example, abatacept works in RA by destroying T cells. Rituximab controls RA by crippling B cell. Adalimumab, etanercept, infliximab, and golimumab work by interfering with the activity of tumor necrosis factor (TNF). Anakinra blocks the action of interleukin-1 (IL-1) in RA. However, only some symptoms and other associated complications of RA patients could be cured by using these biological DMARDs, and the side effects (such as infection, liver damage, reduced ability to make new blood cells, nausea, and pain or swelling at the injection site) also cannot be ignored.

The intimal synovial lining mostly contains two types of synoviocytes: macrophage-like synoviocytes (MLS) and fibroblast-like synoviocytes (FLS) [6]. FLS is much more abundant than MLS and constitutes a central cellular component in the synovium [7]. FLS is an active player in RA joint synovium by promoting synovitis, pannus growth, and cartilage/bone destruction [8]. In RA, FLS secretes a variety of proinflammatory factors (such as TNF-*α*, IL-1, and IL-6) and produces a panel of anti-inflammatory factors (including prostaglandins, vascular endothelial growth factor) in synovium. In addition, FLS also hyperproliferates in RA, mostly due to the reduced apoptosis [9]. As summarized in a recent review [10], it seems that FLS plays a dual role in RA, that is, aggressive “imprinted” behavior and passive response to the inflammatory microenvironment. Therefore, FLS could be considered as a potential target for treatment of RA without affecting the immune system. However, currently, there is no specific FLS-targeted drug for RA treatment.

To date, much has been learnt about microRNAs (miRNAs) since their discovery. The essential regulatory roles of miRNAs in autoimmune and autoinflammatory diseases also have been well studied [11, 12]. However, insufficient information is available for obtaining a systematic and holistic view of whether miRNA dysfunction might play an essential role in relation to FLS in RA. This review therefore focuses on the potential roles played by miRNAs in RA development and maintenance, with emphasis laid on RA FLS. In this review, the role of DICER1, a key synthetase in miRNA biogenesis, in relation to RA FLS will be introduced. All the individual functional miRNAs reported in relation to FLS in RA have been divided into three categories: Wnt signaling, the NF-*κ*B signaling pathway, and epigenetic-related groups. The potential future use of these miRNAs in RA diagnosis and treatment has also been discussed in this review.

### 1.1. The Role of DICER1 in RA FLS

**Figure 1:**
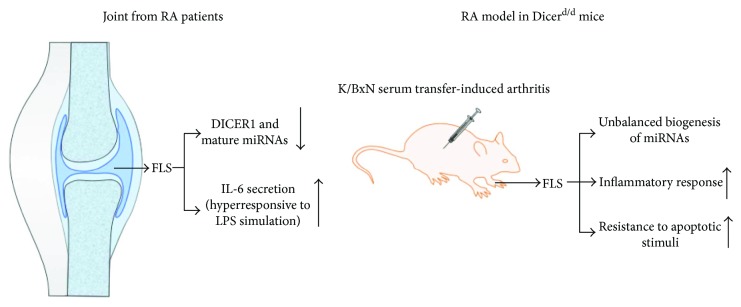
The role of DICER1 in RA FLS.

DICER1, an endoribonuclease from the RNase III family, is essential for miRNA processing. Mutations in DICER1 are associated with several human diseases, mainly different types of cancers [13, 14]. The roles of several individual miRNAs in relation to RA FLS have been reported, but only one study has focused on DICER1 in FLS in RA [15]. Alsaleh et al. showed that both DICER1 and global miRNAs were repressed in FLS from patients with RA [15]; The DICER1 repression led to induction of interleukin- (IL-) 6 production, a well-defined proinflammatory cytokine, in the RA FLS pretreated with lipopolysaccharide (LPS), suggesting that DICER1 repression could promote the inflammation status in FLS from RA patients. A serum-induced arthritis model involving Dicer^d/d^ mice (Dicer1-deficient mice) was used in the same study to further confirm that dysregulated biogenesis of total miRNAs is also along with a hyperresponsive inflammatory reaction in FLS from RA mice. Enhanced resistance to apoptotic stimulation was noted in FLS from both patients with RA and Dicer^d/d^ mice (Figure 1). This study accentuated the essential function of DICER1 in FLS during rheumatic responses, which also highlights the important roles of individual miRNAs in RA FLS.

### 1.2. Wnt Pathway-Related miRNAs in RA FLS

**Figure 2:**
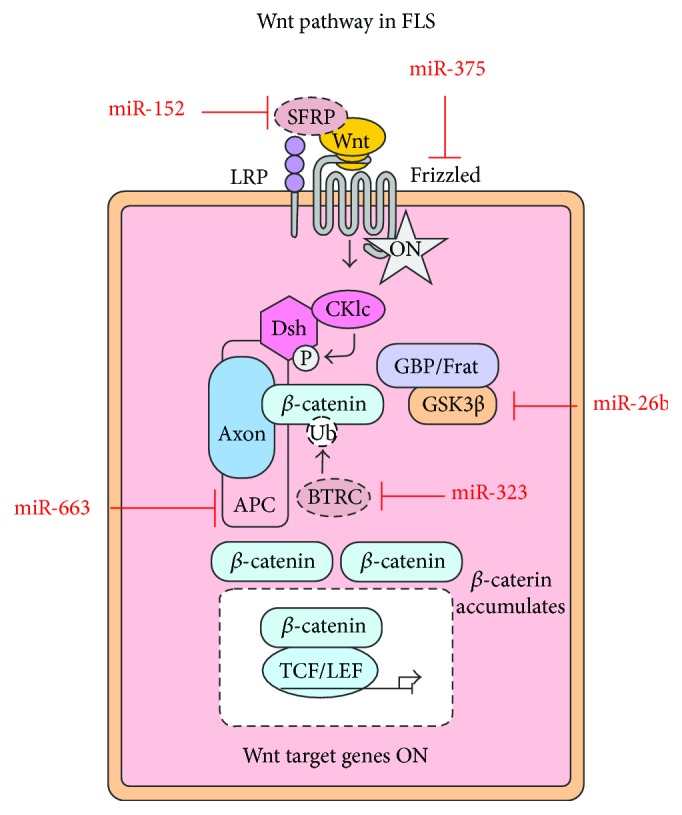
Wnt pathway-related microRNAs in RA FLS.

Recent research has shown that the Wnt signaling pathway plays a pivotal role in FLS during the process of pathogenesis in RA [16]. The Wnt family is a group of glycoproteins that binds to Fz receptors on the cell surface. Subsequently, through the intracellular/endonuclear cascades, the Wnt pathway can regulate several important physiological and pathological conditions, such as skeletal development and cancer, via regulation of the core transcriptional factor *β*-catenin within the nucleus [17]. FLS activation is closely associated with the Wnt pathway in the pathogenesis of RA [18]. To further investigate the upstream regulators of the Wnt pathway in RA FLS would extend our understanding of RA pathogenesis and the related clinical prognosis and therapy. Interestingly, a series of studies have shown that several miRNAs affect RA FLS via interaction with the Wnt signaling pathway. Here, we summarize the research to date on crosstalk between Wnt signaling components and miRNAs in RA FLS and also highlight the relationship between the Wnt pathway and miRNAs during RA initiation and maintenance (Figure 2).

Sun et al. found that miR-26b could induce two key factors of the Wnt pathway, that is, *β*-catenin and cyclin D1, by inhibiting GSK-3*β* expression [19]. Activation of the Wnt/*β*-catenin pathway consequently led to decreased proliferation of RA FLS and promoted FLS apoptosis and the production of proinflammatory cytokines from RA FLS [19]. Using an adjuvant arthritis (AA) rat model, Miao et al. [20] recently showed that increased miR-375 expression inhibited several RA markers (including matrix metalloproteinase [MMP]3 and fibronectin) during the pathogenesis of AA in rats. miR-375 could inhibit the canonical Wnt signaling pathway, and the stabilized form of *β*-catenin blocked the effects of miR-375. Frizzled class receptor 8 (FZD8) was identified as a target of miR-375 in a rat model of AA. FZD8 is also an upstream intriguer of the canonical Wnt pathway. The same group found that another miRNA, miR-663, could induce the canonical Wnt signaling pathway via suppression of Adenomatous polyposis coli (APC) protein expression in FLS [21]. Upregulation of miR-663 also promoted FLS proliferation and the expression of several key disease markers during the development of the RA model. To identify novel functional miRNAs in RA FLS, FLS isolated from the human tumor necrosis factor (TNF) transgenic mouse model were used for miRNA-seq, and FLS from biopsy samples from patients with RA were used for validation [22]. miR-221/222 and miR-323-3p were found to be dysregulated in RA FLS. The researchers showed that miR-323-3p acts as a positive regulator of Wnt signaling in RA FLS by repressing BTRC, an inhibitor of *β*-catenin accumulation [22]. Ectopic expression of miR-152 indirectly induced expression of SFRP4, a negative regulator of the Wnt pathway, by targeting DNMT1 [23]. This is a potential mechanism underlying the antiproliferative effect of miR-152 on FLS. In addition, DNMT1 is an essential DNA methyltransferase, which indicates that DNA methylation may also be another molecular mechanism involved in the modulation of the canonical Wnt pathway in RA FLS.

Recent findings (summarized in Figure 2) involving FLS obtained from patients with RA showed that miRNAs can comprehensively regulate Wnt signaling pathways at several levels (including at the ligand, receptor, and intracellular factor levels and by blocking the Wnt pathway) and regulate the expression of multiple proteins that could potentially promote cell migration and differentiation in the rheumatoid synovium. On the basis of these findings, it is reasonable to conclude that these miRNAs could potentially promote synovial inflammation, pannus formation, and bone and cartilage erosion during RA pathogenesis via regulation of the Wnt signaling pathway. Further studies are required on the potential of these Wnt pathway-related miRNAs as diagnostic, therapeutic, or prognostic markers or targets for RA treatment.

### 1.3. NF-*κ*B Pathway-Related miRNAs in RA FLS

**Figure 3:**
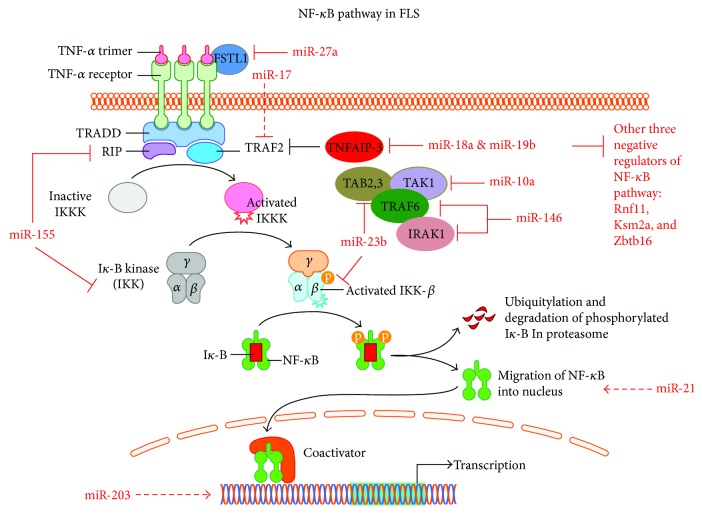
NF-*κ*B pathway-related microRNAs in RA FLS (solid line: direct regulation; broken line: indirect regulation).

RA FLS create an abnormal stromal environment by secreting proinflammatory cytokines and growth factors that stimulate bone/cartilage damage and neovascularization; this environment is pivotal for the development of inflammation [7]. FLS have certain characteristics, such as cancer cell-like features, that are not exhibited by other normal fibroblasts [7]. The NF-*κ*B signaling pathway is a key pathway that contributes to these features in FLS [24]. This finding indicates that the NF-*κ*B pathway-related miRNAs in FLS would also contribute to the pathological behavior of FLS in RA (Figure 3).

Locked nucleic acid (LNA) microarrays have been used to profile global miRNA expression in RA FLS [25]. miR-155 and miR-146a were found to be significantly upregulated in RA FLS, and the roles of these two miRNAs were further validated by other studies [26, 27]. Specifically, miR-146 could directly repress IRAK1 and TRAF6, two important components of the NF-*κ*B pathway. MiR-146a also was elegantly proved as a key regulators of FLS activation and joint pathology in mice arthritis [28]. miR-146a deficiency induced the ability of FLS to support the generation of osteoclasts by controlling the balance of osteoclastogenic regulatory factor receptor activator of NF-*κ*B ligand (RANKL) and osteoprotegerin (OPG). Similarly, miR-155 could also affect the NF-*κ*B pathway via direct targeting of two other essential genes, RIP1 and IKK. Shi et al. [29] found that endogenous expression of miR-27a was significantly repressed in different tissues from patients with RA; samples in which repression was seen included serum, synovium, and FLS. In this study, miR-27a was found to markedly decrease the expression of MMP 2, MMP 9, MMP 13, and Rho family proteins in RA FLS. In addition, miR-27a was found to restrain the migration and invasion of RA FLS by repressing FSTL1, which consequently led to blocking of the NF-*κ*B pathway in RA FLS. miR-21 is overexpressed in the serum of patients with RA [30]. Expression of miR-21 and NF-*κ*B were found to be positively correlated in FLS isolated from an RA rat model, suggesting that miR-21 functions as an activator of the NF-*κ*B pathway in RA FLS. Experimental results proved that miR-21 knockdown could repress expression of the nucleoprotein NF-*κ*B in and proliferation of RA FLS. In contrast, ectopic expression of miR-21 in FLS was found to increase nucleoprotein NF-*κ*B levels and cell proliferation rates, further validating that miR-21 could promote FLS proliferation by facilitating NF-*κ*B nuclear translocation, consequently affecting the function of the NF-*κ*B pathway in RA FLS. The proinflammatory cytokine IL-17 can facilitate the progress of inflammatory autoimmune diseases, which leads to downregulation of miR-23b in human FLS [31]. miR-23b is repressed by IL-17 in the inflamed synovium of humans with RA and in mouse models of RA. miR-23b also suppresses IL-17, TNF-*α*, and IL-1*β*-induced NF-*κ*B activation and inflammatory cytokine expression by targeting TAB2, TAB3, and IKK-*α* and, consequently, represses autoimmune inflammation. Thus, the IL-17-miR-23b-NF-*κ*B axis promotes autoimmune pathogenesis in RA FLS. Mu et al. [32] reported a novel NF-*κ*B/YY1/miR-10a axis that activates the production of NF-*κ*B pathway-mediated inflammatory cytokines. This regulatory circuit can also promote the proliferation and migration of RA FLS. miR-203 was found to be dependent on NF-*κ*B activity for modulating IL-6 expression in RA FLS [33] and was identified as a novel regulator of IL-6 in these cells in RA. As IL-6 is an upstream modulator of NF-*κ*B, miR-203 could indirectly affect the NF-*κ*B pathway in RA FLS. However, the direct target of miR-203 could not be identified on using a miRNA-prediction algorithm.

miR-17-92 is an essential miRNA cluster in RA FLS. miR-17, one of the miRNA members from this cluster, was found to be expressed at basal levels in serum, FLS, and synovial tissues from a patient with RA, as well as in the serum and joints of rats with AA [34]. RNA-seq and ingenuity pathway analyses were used to identify potential targets and miR-17-related pathways in RA FLS. Destabilization of TRAF2 by miR-17 leads to inhibition of TNF-*α*-induced NF-*κ*B p65, c-Jun, and STAT3 nuclear translocation and the production of several essential proinflammatory cytokines and enzymes, including IL-6, IL-8, MMP-1, and MMP-13 in human RA FLS [34]. miR-18a, another miRNA derived from the miR-17-92 cluster, promotes inflammation development and cartilage destruction through interaction with the NF-*κ*B signaling pathway in RA FLS [35]. TNF-*α*-induced protein 3 (TNFAIP-3), an NF-*κ*B signaling pathway inhibitor, was identified as a direct target of miR-18a in RA FLS. Repression of the NF-*κ*B pathway by miR-18a also leads to upregulation of matrix-degrading enzymes and mediators of inflammation in RA FLS [35]. Gantier et al. [36] showed that positive regulation of NF-*κ*B signaling by miR-19b involves the synergistic suppression of a group of repressors of NF-*κ*B signaling. miR-19b promotes the inflammatory activation of RA FLS, highlighting the potential physiological importance of this miRNA in RA pathology.

Taken together, all these studies indicate that interactions between the NF-*κ*B pathway and associated miRNAs play a major role in the pathogenesis of RA. Thus, these miRNAs act as a “valve” to control the NF-*κ*B pathway in RA FLS; further studies are required to evaluate these potential diagnostic and therapeutic targets for RA treatment.

### 1.4. DNA Methylation-Related miRNAs in RA FLS

Epigenetics is an emerging and promising research area since epigenetic alterations are both heritable and acquired throughout life, and this field is ideal for bridging analyses of environmental and genetic contributions to risk of disease. The most well-studied epigenetic regulators include DNA methylation, histone modification, microRNAs, and so on. While many epigenetic studies on autoimmune diseases have focused on epigenetic regulators in immune cells, perhaps the most well-established research in RA relates to the stromal element, primarily, FLS from the inner layer of the synovial membrane surrounding the joints [7]. The existence of imprinted phenotypes of FLS suggests epigenetic alterations of these cells, which could partly explain the variability in RA severity [37]. The biology of RA FLS remains incompletely understood and currently RA treatment does not include any FLS-targeted agents. Investigation of interaction between microRNAs and other epigenetic regulators, such as DNA methylation, of RA FLS could provide an important approach for identifying novel diagnostic and therapeutic agents [38].

**Figure 4:**
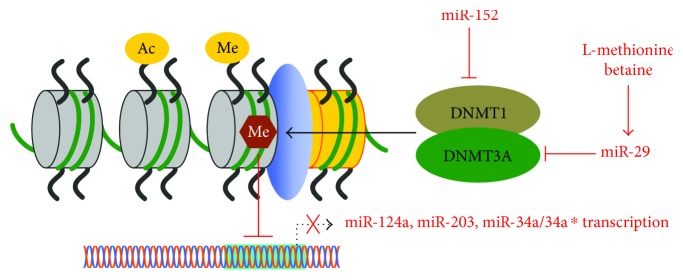
Epigenetics related microRNAs in RA FLS.

The genomic DNA in RA FLS is generally hypomethylated, which might be associated with some aggressive “tumor-like” phenotypes [39, 40]. Therefore, researchers attempt to remethylate these cells with methyl donors (l-methionine and betaine) and investigate miR-29, as this miRNA could target the DNA methyltransase DNMT3A. miR-29 might repress the remethylation effect of betaine. Betaine has beneficial effects on the activated phenotype of RA FLS; so clinical trials with betaine could provide promising therapeutic options [39]. Zhou et al. found that 5-aza-2′-deoxycytidine (5-AzadC) represses the methylation of miR-124a in RA FLS and restores RA FLS proliferation and TNF-*α* expression [41], suggesting that DNA methylation of a single gene could regulate inflammatory cytokine secretion and RA development. Another similar study performed on DNA demethylation with 5-azaC showed increased miR-203 expression [33]. Overexpression of miR-203 led to significantly increased levels of MMP-1 and IL-6; endogenous expression of miR-203 was regulated by DNA methylation in RA FLS. Importantly, it also showed that the induction of MMP-1 and IL-6 by miR-203 was NF-*κ*B pathway dependent, implying potential interaction between DNA methylation and the NF-*κ*B pathway in RA FLS. Miao et al. showed that miR-152 was significantly repressed in a rat model of AA and that ectopic expression of miR-152 in FLS could downregulate DNMT1. miR-152-mediated DNA methylation could activate the canonical Wnt pathway in RA; therefore, epigenetic DNA modifications may provide a target for RA treatment [23]. A study involving methylation of the promoter of miR-34a/34a^∗^ showed that transcription of miR-34a/34a^∗^ was induced on treatment with DNA demethylation agents [42]. Enforced expression of miR-34a^∗^ led to an increased rate of FasL- and TRAIL-mediated apoptosis in RA FLS. These studies provide evidence of methylation-specific downregulation of proapoptotic miR-34a^∗^ in RA FLS. An understanding of the manner in which epigenetic changes contribute to the function of individual miRNAs and to RA pathogenesis has been obtained in recent years (Figure 4). Further research is required to determine the interplay between different epigenetic modifications. In addition, more functional studies need to be performed to comprehend the mechanisms underlying epigenetic regulations in FLS that contribute to RA pathogenesis.

## 2. Conclusion and Future Perspective

Previous studies in a mouse arthritis model indicated that at least two cellular mechanisms are involved in the pathogenesis of RA [43], which further highlights the important role played by FLS in different stages of RA. Because of their extensive participation in mediating extracellular interactions and intracellular crosstalk, FLS represent an essential target for novel therapeutic approaches for RA [43].

Recent studies suggest that miRNA dysfunction plays a pivotal part in RA FLS (Table 1). The manner in which miRNAs are deregulated in RA could be manifold, ranging from germline loss or gene amplification of miRNAs to their transcriptional deregulation, possibly by epigenetic modifications or activation. The interaction between two well-established signaling pathways, the Wnt and NF-*κ*B signaling pathways, and related miRNAs could also result in RA maintenance and development. In addition, the research of complicated relationship between miRNAs and epigenetic factors in FLS contributing to the pathogenesis of RA has increased in recent years. Among all known epigenetic modifications, studies have focused on interaction between DNA methylation and miRNAs in RA FLS. To understand the mechanisms underlying the contribution of epigenetic factors in FLS to RA pathogenesis, the interplay between other epigenetic modifications, such as histone and RNA modifications, and miRNAs in RA FLS would need to be determined.

The candidate RA miRNAs identified to date in FLS have already improved our understanding of the molecular mechanisms underlying RA. By identifying functional miRNAs in FLS from patients with RA, several target genes can be simultaneously manipulated by modulating individual miRNAs. miRNA expression profiling in FLS from patients with RA could be potentially useful for identifying biomarkers for assessing disease phenotype, activity, progression, or treatment response. Therefore, a greater number of individual RA-related miRNAs in RA FLS should be investigated and evaluated. Furthermore, the off-target effect of individual miRNAs within FLS or in other cellular players in the RA synovium could also be considered when a particular miRNA is considered as a potential therapeutic target for RA.

## Figures and Tables

**Table 1:** The summary of individual miRNAs in RA FLS.

miRNA	Targets	RA	Pathways	Reference
miR-26b	GSK-3P	Repression	Wnt	[19]
miR-375	FZD8	Repression	Wnt	[20]
miR-663	APC	Promotion	Wnt	[21]
miR-323	BTRC	Promotion	Wnt	[22]
miR-152	DNMT1	Repression	Wnt	[23]
miR-146	IRAKI, TRAF6	Promotion	NF-*κ*B	[26, 28]
miR-155	RIP1JKK	Promotion	NF-*κ*B	[27]
miR-27a	FSTL1	Repression	NF-*κ*B	[28]
miR-21	NF-*κ*B nuclear translocation	Promotion	NF-*κ*B	[29]
miR-23b	TAB2, TAB3, and IKK-a	Repression	NF-*κ*B	[22]
miR-lOa	IRAK4, TAK1 and BTRC	Promotion	NF-*κ*B	[32]
miR-203	Not identified	Promotion	NF-*κ*B	[33]
miR-17	TRAF2	Repression	NF-*κ*B	[34]
miR-18a	TNFAIP-3	Promotion	NF-*κ*B	[35]
miR-19	Rnfll, Kdm2a, Tnfaip3, Zbtbl6	Promotion	NF-*κ*B	[36]
miR-29	DNMT3a	Repression	DNA methylation	[39]
miR-152	DNMT1	Repression	DNA methylation	[23]
miR-124/203/34a/34a^∗^	Regulated by DNA methylation	Promotion	DNA methylation	[33, 41, 42]

## Abbreviations

miRNA: MicroRNA
UTR: Untranslated region
FLS: Fibroblast-like synoviocyte
RA: Rheumatoid arthritis
RF: Rheumatoid factor
MRI: Magnetic resonance imaging
DMARD: Disease-modifying anti-RA drugs
TNF: Tumor necrosis factor
LPS: Lipopolysaccharide
IL: Interleukin
AA: Adjuvant arthritis
MMP: Metalloproteinase
NF-*κ*B: Nuclear factor-kappa B
TAB: TGF-*β*-activated kinase 1/MAP3K7 binding protein
IKK: Inhibitor of nuclear factor kappa B kinase
TRAF2: TNF receptor associated factor 2
TNFAIP-3: Tumor necrosis factor a-induced protein 3
5-azaC: 5-Aza-2′-deoxycytidine
SCID mice: Severe combined immunodecifient mice.
